# An Ultra-Low-Power Analog Multiplier–Divider Compatible with Digital Code for RRAM-Based Computing-in-Memory Macros

**DOI:** 10.3390/mi14071482

**Published:** 2023-07-24

**Authors:** Yiming Yang, Shidong Lv, Xiaoran Li, Xinghua Wang, Qian Wang, Yiyang Yuan, Sen Liang, Feng Zhang

**Affiliations:** 1 School of Integrated Circuits and Electronics, Beijing Institute of Technology, Beijing 100081, China; yangyiming@bit.edu.cn (Y.Y.); 3220225265@bit.edu.cn (S.L.); 89811@bit.edu.cn (X.W.); 3120210707@bit.edu.cn (Q.W.);; 2 BIT Chongqing Institute of Microelectronics and Microsystems, Chongqing 401332, China; 3 Yangtze Delta Region Academy of Beijing Institute of Technology, Jiaxing 314000, China; 4 Institute of Microelectronics, Chinese Academy of Sciences, Beijing 100029, China; yuanyiyang@ime.ac.cn (Y.Y.); zhangfeng_ime@ime.ac.cn (F.Z.)

**Keywords:** CMOS, computing-in-memory, current mirror, edge computing, multiplier–divider

## Abstract

This manuscript presents an ultra-low-power analog multiplier–divider compatible with digital code words, which is applicable to the integrated structure of resistive random-access memory (RRAM)-based computing-in-memory (CIM) macros. Current multiplication and division are accomplished by a current-mirror-based structure. Compared with digital dividers to achieve higher precision and operation speed, analog dividers present the advantages of a reduced power consumption and a simple circuit structure in lower precision operations, thus improving the energy efficiency. Designed and fabricated in a 55 nm CMOS process, the proposed work is capable of achieving 8-bit precision for analog current multiplication and division operations. Measurement results show that the signal delay is 1 μs when performing 8-bit operation, with a bandwidth of 1.4 MHz. The power consumption is less than 6.15 μW with a 1.2 V supply voltage. The proposed multiplier–divider can increase the operation capacity by dividing the input current and digital code while reducing the power consumption and complexity required by division, which can be further utilized in real-time operation of edge computing devices.

## 1. Introduction

Dividers are critical components in many applications. In algorithms that involve large-scale computation such as convolutional neural networks (CNN), division operations are required during normalization processes and other related algorithms. Intelligent edge computing equipment that involves image processing often requires several steps such as image acquisition, image pre-processing, neural network operation, and result output. In addition to the normalization process in the neural network algorithm that requires division, some image preprocessing algorithms also require division before the data are input into the neural network, such as histogram equalization, batch normalization (BN), mean filtering, etc.

**Figure 1:**
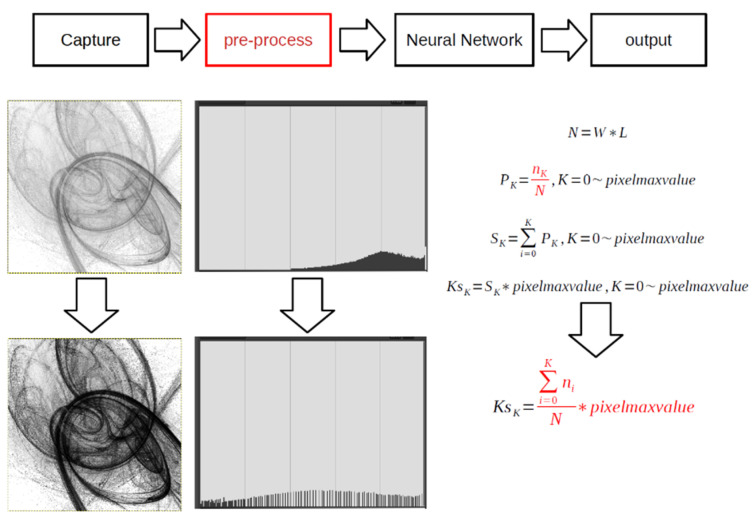
The effect and mathematical equations of the histogram equalization algorithm. Division is inevitably applied for the equation marked in red.

**Figure 2:**
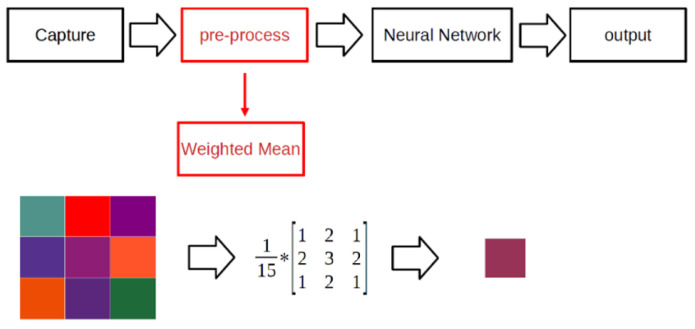
The weighted mean algorithm, which is another image preprocessing algorithm. Division is inevitably used in the mean process.

Histograms are commonly used to represent the distribution of brightness levels in an image. By extending the histogram to a wider range, the contrast of the image can be easily enhanced, which in turn can improve the accuracy of network-based reasoning. This process involves integrating the pixel values for each brightness level and performing simultaneous multiplication and division by *N* to normalize the values. Figure 1 illustrates the effect and mathematical equations of the histogram equalization algorithm. Adding histogram equalization to the pre-processing stage can enhance overall accuracy [1]. The purpose of batch normalization (BN) is to standardize the output of the middle layer of the CNN. During the BN process, the sample mean and variance of the layer inputs are computed and adjusted. BN can stabilize and accelerate the training of the neural network and facilitate better gradient flow throughout the network. Mean filtering is a technique that involves smoothing by reducing noise and eliminating high-frequency components from an image. It operates on each pixel, multiplying and dividing its matrix by coefficients and summing the resulting products. This process requires simultaneous multiplication and division of an arbitrary and variable number. The weighted mean algorithm is shown in Figure 2. Mean filtering can effectively filter out additive Gaussian noise in the image and perform a mean pooling operation between each layer of the neural network, thereby improving its accuracy [2].

With the development of CNNs, the huge amount of complicated computation severely limits the application of CNNs in edge intelligent Internet of Things (IoT) devices due to the limitation of power consumption. Non-volatile computing-in-memory (nvCIM) architecture based on memristors for building up neural networks effectively breaks the bottleneck of the memory wall [3]. Resistive random-access memory (RRAM)-based CIM accelerators have significant advantages in edge computing due to the potential to perform CNN computations in an energy-efficient manner. RRAM can accelerate CNN computations and export the result of matrix–vector multiplication in an analog current form, making it a promising technology for edge devices. While multiplication and addition have been extensively researched for RRAM-based CIM, division has been mostly avoided due to the difficulty of implementation. Digital dividers can be utilized for computing, but additional analog-to-digital converters (ADC) are needed for data transmission. Furthermore, traditional digital division suffers from a higher power consumption, a larger area, and a lower speed. Analog dividers can achieve high speed while reducing the power consumption and circuit complexity [4].

Conventional analog multipliers and dividers can be mainly implemented through two approaches. One is to use current-mode amplifiers [5,6,7,8], such as current-following transconductance amplifiers (CFTA) [6], current-controlled conveyors (CCC) [7,8], etc. These active amplifiers provide a higher gain, which leads to more precise calculation results. However, these amplifiers may increase the power consumption and are commonly implemented using bipolar transistors that require a high supply voltage, generally requiring a negative supply voltage. Another kind of multiplier and divider is based on the unique square characteristic of MOSFET [9,10,11,12], or the exponential characteristic of the weak inversion region [13,14,15,16], which can achieve a higher bandwidth, smaller size and lower power consumption. However, due to the limited working area of the transistor, the operation range is narrow. In addition, the output is an approximate value, and the accuracy at both ends of the operation range is slightly worse. Recently, a new pulse-based analog divider was proposed, which can use a digital code word as output and is compatible with digital systems with high precision [17]. However, it suffers from a large area, a high power consumption, and a relatively slow speed. As CMOS technology continues to develop, the speed and accuracy of current mirrors are becoming higher, providing a possibility for the implementation of new analog multiplication and division circuits.

This manuscript proposes an analog multiplier–divider compatible with digital code words that can be used for real-time division operations of edge devices and to improve energy efficiency. The current can be conveniently and precisely multiplied or divided through the current mirror. The multiplication and division of the current can be completed only by controlling the multiples at both ends of the current mirror. The whole process requires no approximation and can satisfy a large range of inputs and outputs with a high accuracy. At the same time, only two transistors have continuous current in the whole circuit, resulting in an increased efficiency. The circuit size required to perform 8-bit operations is also small. Therefore, the proposed small size and ultra-low-power multiplier–divider is suitable for real-time operations in an RRAM-based CIM macro to improve the computing capacity.

The remainder of this paper is organized as follows. Section 2 describes the circuit implementation details of the proposed multiplier–divider. The measurement results are discussed in Section 3. A conclusion is provided in Section 4.

## 2. Circuit Implementation

**Figure 3:**
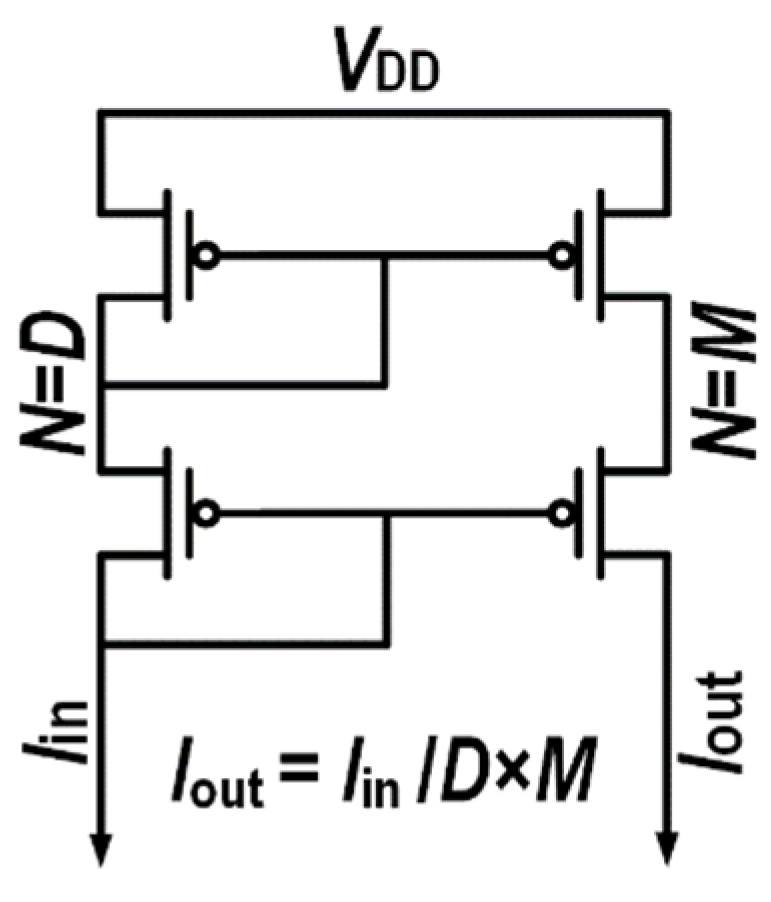
The basic structure of current multiplication and division using current mirror. The current multiples on the input side and output side are *D* and *M*, respectively.

The output current can be multiplied or divided by controlling the current mirror multiples. As shown in Figure 3, the output current can be calculated in Equation (1).

(1) $$I_{out}=\frac{M}{D}\times I_{in}$$

It is difficult to change the ratio (*D* and *M*) of the current mirror after fabrication. Therefore, for practical use, it is necessary for both the divisor and dividend to be variable, allowing for continuous adjustment or at least covering all integer values.

**Figure 4:**
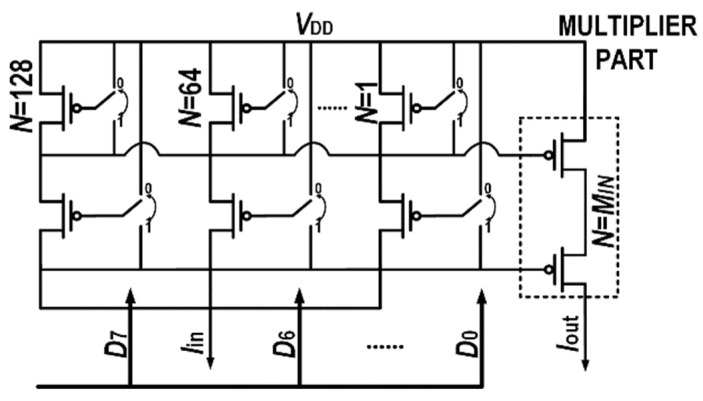
The schematic of the divider part. The divisor *D* can be changed from 0 to 255 by switches controlled by digital code *D*_0_~*D*_7_.

Figure 4 shows the current mirror with any integer multiple. Only the transistor gate is connected to the circuit, reducing the load capacitance of the input stage. To ensure accuracy, a two-stage cascode current mirror is employed. The input current mirrors are divided into eight groups with multiples of 1, 2, 4, 8, 16, 32, 64, and 128, respectively. As a result, a total of 255 times input current can be connected, performing 8-bit operation. When *N* = *M*_IN_ = 1, the multiple of the output current mirror is 1. The output current is *I*_in_, divided by the current mirror multiple on the input side. When the divisor input is high, the gates of the input-side current mirrors are connected to the output-side transistor gates and to the input-side transistor drains. When the divisor input is low, the gates will be connected to *V*_DD_. This allows calculations of all 8-bit binary multiples, thus completing the division between the analog input current and the 8-bit divisor. When the input current is an integer multiple of the discrete amplitude of the unit current, the output current is the multiple of the operation result of the integer of the same unit current.

Meanwhile, the output is a continuous value since the proposed structure retains the characteristics of an analog circuit. In this case, the multiple will not be rounded to the decimal part, if any, which can meet the rounding requirements often required in image processing algorithms. The circuit is also naturally compatible with division by 0. When *D*_IN_ is zero, all the gates of the input-side transistors will be connected to *V*_DD_, while the gates of the output-side transistors will be directly connected to the drains at the input side. There will be a large voltage drop due to the high turn-off resistance with current input. However, transistors farther from the power supply will have higher threshold voltages and higher turn-off resistance due to substrate bias effects for the cascode structure. As a result, most of the voltage drops will occur on this transistor, while transistors closer to the power supply will have less voltage drops. The gate of the transistor that is farther from the power supply on the output side is almost grounded and turned on, while the gate voltage of the transistor closer to the power supply is almost the power supply voltage. At this time, the total current is the current of the transistor with the smaller current, and the output is almost zero, which can simulate the effective bit return of 0 after the division overflow. 

**Figure 5:**
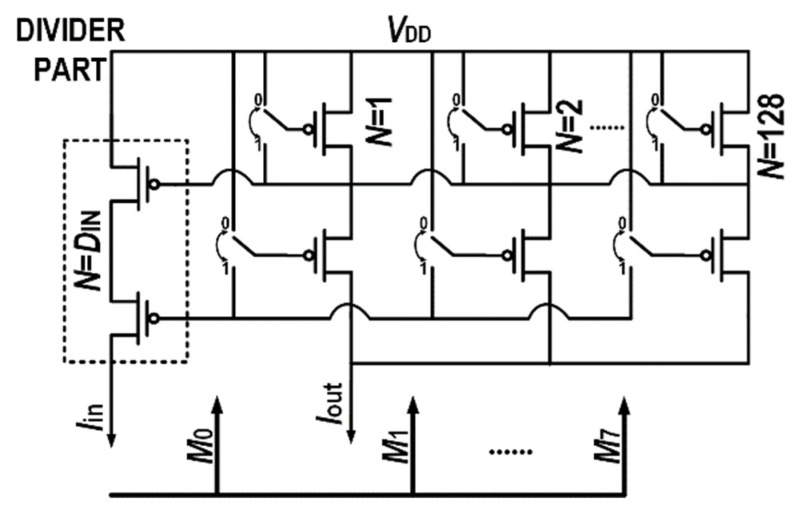
The schematic of the multiplier part. The multiple *M* can be changed from 0 to 255 by switches controlled by digital code *M*_0_~*M*_7_.

Both mentioned operations require one multiplication and one division. Therefore, completing the multiplication simultaneously will increase the working efficiency of the system. Multiplication can be performed by controlling the multiple of the current mirror at the output side. As shown in Figure 5, the multiplier part is similar to the divider part, which is also composed of cascode current mirrors. The 8-bit input *M*_IN_ controls the connection of eight groups of gates. When the multiplier input is high, the gates of the output-side current mirrors will be connected to the gates of the input-side transistors. When the multiplier input is low, the gates will be connected to *V*_DD_. By changing the multiple of the current mirror at the output side, the result of the division from the divider part can be multiplied by the 8-bit *M*_IN_ input. The final output current can be calculated in Equation (2), enabling the multiplication and division to be performed in a single step.

(2) $$I_{out}=\frac{M_{IN}}{D_{IN}}\times I_{in}$$

Since the multiplier part and the divider part have the same analog circuit characteristics, the output is the result of direct operation between the division result and the multiplier. Compared with the integer division followed by multiplication in digital circuits, the proposed work will not produce errors by discarding the decimal places directly. It can naturally guarantee the accuracy of the final result without any rounding during the operation, and if the result is also a decimal, the result will not be discarded. Similarly, when the multiplier input is 0, all the gates of transistors on the output side are connected to the power supply, that is, all the gates of transistors are turned off, which can ensure a very strong zero current output.

**Figure 6:**
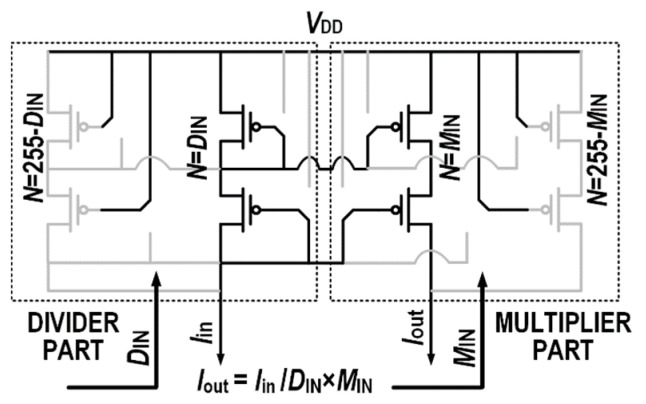
The schematic of the proposed multiplier–divider.

**Figure 7:**
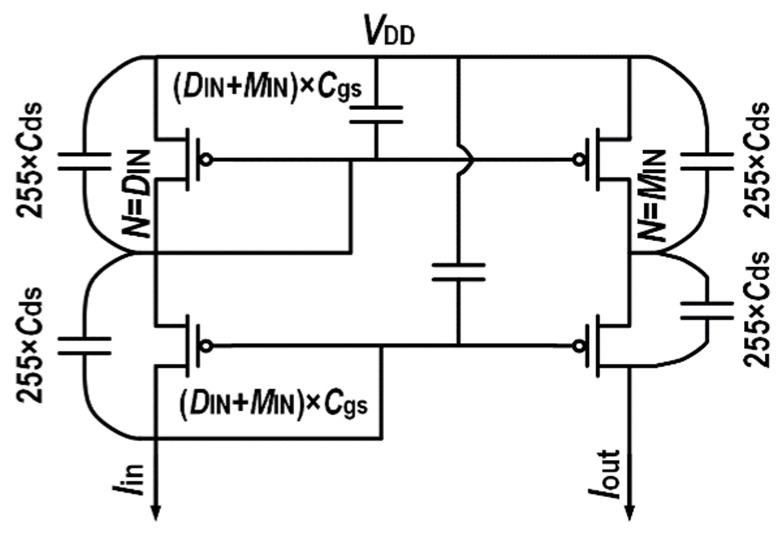
The parasitic capacitance of the divider.

A schematic and the complete operation process of the proposed multiplier–divider are shown in Figure 6. The W/L of the single unit (when N = 1) PMOS in the current mirror is 4 μm/300 nm. When the input divisor is given at *D*_IN_ and the input multiplier is given at *M*_IN_, the current mirrors on both sides are connected to *D*_IN_ and *M*_IN_, respectively, while 255-*D*_IN_ and 255 min are disconnected, respectively. The input current flows through the current mirror to complete the multiplication and division operation. Figure 7 shows the parasitic capacitor connected during the operation. The input capacitance is (*D*_IN_ + *M*_IN_) × *C*_gs_ and 255 × *C*_ds_. Since only current operation is used, capacitor charging and discharging only require a relatively small voltage fluctuation, which can result in higher speeds and a lower power consumption than voltage operation. The output capacitance is 255 × *C*_ds_. With the cascode current mirror load, the gain is relatively high with higher operation speeds.

Furthermore, the current mirror exhibits temperature-independent characteristics. Even if the temperature and threshold voltage change, as long as the transistors are matched, the operation precision will not be affected. In addition, the average current of the transistors on both sides of the current mirror is consistent, and the mismatch caused by serious unbalanced temperature distribution will not occur. Therefore, the accuracy only depends on the mismatch of the transistor caused by the deviation in fabrication, which is fixed after fabrication and can be calibrated.

**Figure 8:**
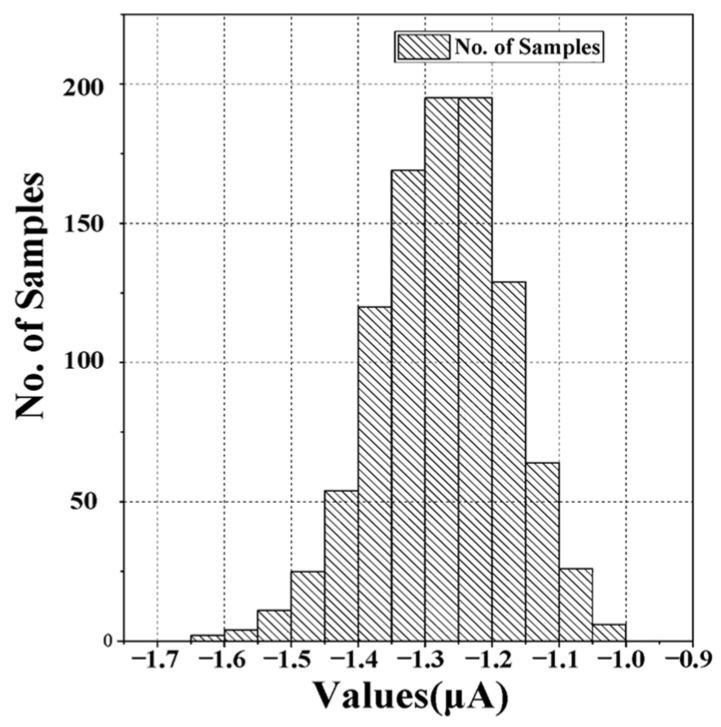
Monte Carlo simulation of the divider.

The Monte Carlo simulation results shown in Figure 8 demonstrate the performance of the divider circuit. The simulation was conducted with a dividend of 255 (input current of 2550 nA), a divisor digital input of 2 (10 b), and a multiplier of 1. The deviation from the ideal value is only 0.31 pA, with an ideal value of 1.275 nA. The 2σ error was ±193.7542 nA, which means that the error was within this range for more than 95% of the cases. These results show that the proposed circuit can provide accurate division with a high precision.

## 3. Measurement Results

**Figure 9:**
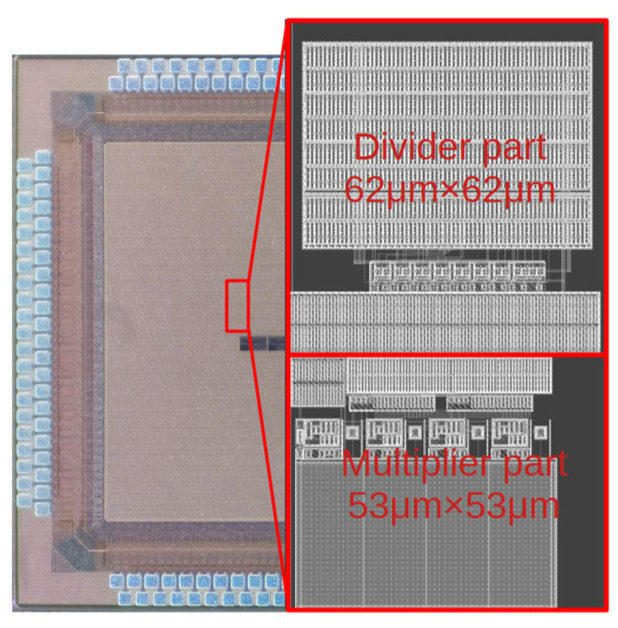
Die micrograph of the fabricated multiplier–divider.

Designed and implemented in a 55 nm ultra-low leakage CMOS process, the die micrograph of the proposed multiplier–divider is shown in Figure 9. The analog current operations enable the circuit to achieve 8-bit precision. The operating bandwidth is 1.4 MHz, and the signal delay for performing 8-bit operation is 1 μs. The maximum power consumption is lower than 6.15 μW under 1.2 V power supply.

**Figure 10:**
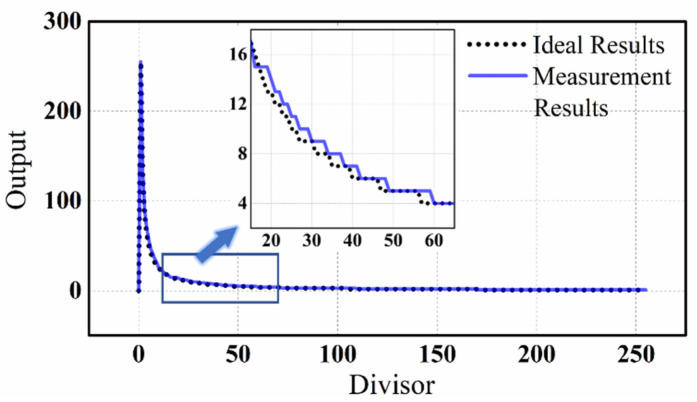
The measured output of the divider and the corresponding ideal division result. The dividend is 255. The *x*-axis represents the divisor, which is 0~255, and the *y*-axis is the division result. The measurement result is the current output after 8-bit analog to digital conversion. The LSB of the ADC is 10 nA and the ideal result is rounded after calculation.

**Figure 11:**
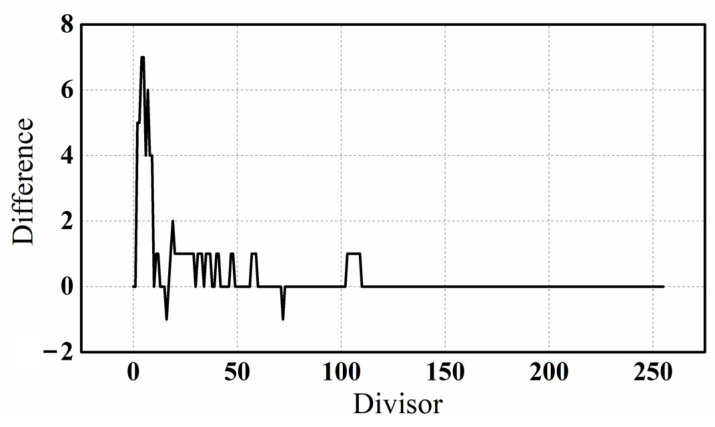
The subtraction between the ideal results and the measurement results of the divider.

The measurement results and ideal results of the analog divider are compared in Figure 10. The dividend used was binary 11111111, which is equivalent to decimal 255, and divisors ranging from 1 to 255 were used. The ideal result is the integer result after the ideal division is rounded, while the measurement result is the integer result corresponding to the analog current. When dividing by 0, the measured output is 0, and ideally the output will generate an error in the operation. The two curves show a slight divergence when the divisor is small, but as the divisor increased, the two curves become almost identical. Figure 11 shows the difference between the two curves in Figure 10. When the divisor is small, the absolute difference between the ideal and measured results can be up to 7. However, after the divisor reaches 25, the difference does not exceed 1.

Table 1 summarizes and compares the measurement performances of the implemented divider with previous state-of-the-art works. It can be found that the divider with CMOS characteristics can achieve higher speeds and a lower power consumption. The pulse-based divider in reference [17] has a higher output bit-width and is compatible with digital code words. However, it cannot perform multiplication, and suffers from a high power consumption and a large chip size. The resistor-based divider in reference [18] is also accurate. However, there is no advantage in power consumption and operation speed, since a separate amplifier chip is applied. Our proposed multiplier–divider exhibits the lowest peak power consumption, a relatively wide data range, and guaranteed, stable accuracy for all signals over the full data range, while is insensitive to temperature, making it compatible with digital code words.

## 4. Conclusions

An ultra-low-power, high-precision, and small-size analog multiplier–divider is presented in this manuscript. It is compatible with digital code words for RRAM-based computing-in-memory, and is capable of performing operations within the range of 8-bit integers. The fractional part of the middle process of multiplication and division can continue to participate in the operation. Designed and fabricated in a 55 nm CMOS process, the measurement results show that the maximum power consumption of the multiplier–divider is less than 6.15 μW under a 1.2 V power supply, and it can operate at a frequency of up to 1 MHz. The proposed work can offer an efficient and accurate solution for multiplication and division operations in various applications, which is suitable for real-time operation in RRAM-based CIM macros, thus improving the computing capacity, and can be utilized in real-time operation of edge computing devices.

## Figures and Tables

**Table 1:** Performance summary with the state-of-the art works.

	[5]	[11]	[12]	[17]	[18]	This Work
Process	0.18 μm CMOS	0.5 μm CMOS	0.18 μm CMOS	0.35 μm CMOS	RRAM	55 nm CMOS
Working Mode	Current	Current	Current	Pulse	Voltage	Current
3 dB Bandwidth	59.7 MHz	18 MHz	62 MHz	/	/	1.4 MHz
Delay Time	/	/	/	/	0.5 ms	1 μs
Multiplier	Yes	Yes	Yes	No	Yes	Yes
Power Consumption	75 μW	120 μW	144 μW	<440 μW	/	<6.15 μW
Accurate or Approximate	Approximate	Approximate	Approximate	Accurate	Accurate	Accurate
Data Width or Range	1~10 μA	20 μA	1~20 μA	0.1~1 V, 12 bit	/	8-bit (256) (0~2550 nA)
Compatible with Digital	No	No	No	Yes	No	Yes
Chip Area	800 μm^2^	0.021 mm^2^	4050 μm^2^	0.25 mm^2^	/	6600 μm^2^

## Data Availability

All the data are contained within this article.
